# Liver Abscess due to *Streptococcus pneumoniae*: A Clinical Rarity

**DOI:** 10.1155/2020/1572023

**Published:** 2020-06-02

**Authors:** Srujana Mohanty, Manas Kumar Panigrahi

**Affiliations:** ^1^Department of Microbiology, All India Institute of Medical Sciences, Bhubaneswar-751019, Odisha, India; ^2^Department of Gastroenterology, All India Institute of Medical Sciences, Bhubaneswar-751019, Odisha, India

## Abstract

We report a case of pyogenic liver abscess caused by a rare causative agent, *Streptococcus pneumoniae*. A 45-year-old man with underlying uncontrolled diabetes mellitus who had stopped taking his daily dose of insulin since the last 4 days, presented with pain in the abdominal area of one-day duration. Upon his admission to achieve diabetic control, a routine ultrasound examination of abdomen revealed incidentally, a large abscess in the left lobe of the liver with impending rupture. Culture of the ultrasound-guided liver aspirate pus yielded pure growth of a penicillin-susceptible *S. pneumoniae* isolate. After 4 weeks of parenteral ceftriaxone therapy along with intensive regimen for diabetic control, the liver abscess became resolved, and the patient improved and was discharged with no residual infection or recurrence at four months and at one-year follow-up. A review of relevant literature related to *S. pneumoniae* liver abscess revealed a mention of such entity only on 4 previous occasions. The present case highlights an important though rare manifestation of *S. pneumoniae* infection and emphasizes the need to establish an early diagnosis of *S. pneumoniae* infection for improved patient survival and favourable outcome.

## 1. Introduction

Pyogenic liver abscess (PLA) is an important clinical entity in the general population with a significant mortality rate in both developing and developed countries [1, 2]. It is a particularly serious infection that can prove fatal if not treated promptly. The annual incidence of PLA varies from 2.3 to 15.45 cases per 100,000 person-years and found more in men than in women [1–3]. Hepatobiliary causes such as cholelithiasis, cholangitis, and obstructive malignancy affecting the biliary tree, biliary strictures, or congenital anomalies account for approximately 40%–60% of PLAs [4, 5]. Other causes include perforated bowel or appendicitis, dental infections, systemic sepsis, infection of contiguous structures, ventriculoperitoneal shunt, blunt trauma, and foreign bodies [4, 5]. Cryptogenic origins sometimes account for up to 80% of cases [5].

The common causative agents implicated are *Streptococcus milleri*, *Klebsiella pneumoniae*, and *Escherichia coli* [1, 2, 5, 6]. *Streptococcus pneumoniae,* a gram-positive bacteria and a commensal nasopharyngeal flora, is a major human pathogen responsible for millions of death and significantly more invasive infections each year worldwide [7]. However, it has been implicated as a causative agent of PLA extremely rare in the literature [8–11]. We report a case of pyogenic liver abscess due to *S. pneumoniae* in an adult patient, the timely recognition of which saved the patient from undergoing a potentially fatal course.

## 2. Case Report

A 45-year-old man with underlying uncontrolled diabetes mellitus, who had stopped taking his daily dose of insulin since the last 4 days, presented with pain in the right abdominal area of one-day duration. He had no fever or any other significant systemic symptoms. He was a known alcoholic, but he had stopped consuming alcohol since the last 4 months. He had no other relevant medical history, including hypertension, history of contact with tuberculosis, or any urinary or bowel complaints. He gave no history of dental extraction or dental manipulation, no surgical history, and no history of bronchoscopy or any respiratory tract manipulation in the recent past. On admission to achieve diabetic control, physical examination revealed a thin-built man (body weight 49 kg) who was awake and oriented to time, place, and person but appeared lethargic and pale. Vital signs included a lowered body temperature of 36.5°C, blood pressure of 126/76 mmHg, pulse of 62/min, and a regular respiratory rate of 18/min. No evidence of dental caries, periodontitis, or any other oral lesions were found. Abdominal examination revealed a soft, nontender, and slightly distended abdomen with no apparent hepatomegaly or splenomegaly.

### 2.1. Laboratory Investigations

**Figure 1:**
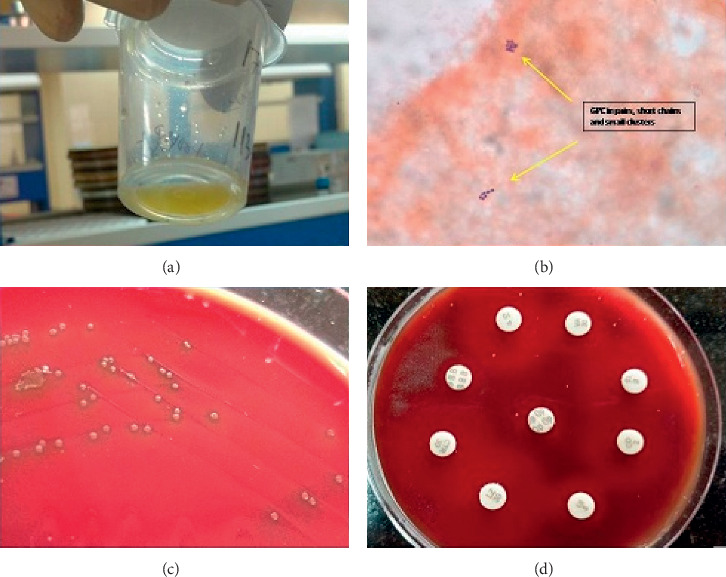
Liver aspirate showing (a) brownish yellow liquid pus and (b) Gram-positive cocci (GPC) arranged in pairs, short chains, and small clusters (1000x). (c) Growth of *S. pneumoniae* on blood agar plate and (d) susceptibility of *S. pneumoniae* to various antibiotics by the disc-diffusion test.

Blood tests revealed an elevated alanine aminotransferase level of 103 IU/L and slightly elevated aspartate aminotransferase level of 44 IU/L, but normal alkaline phosphatase level of 178 IU/L. Hemogram reports were within normal limits except slight leukocytosis with a total leucocyte count of 11.1 × 10^9^/L (66% polymorphs, 21% lymphocytes, and 9% eosinophils), total red blood cell count of 4.9 × 10^12^/L, platelet count of 324 × 10^9^/L, and haemoglobin level of 129 g/L. Blood metabolic panel suggested an extremely poor diabetes control with a high random blood sugar level of 299 mg/dL, fasting blood glucose level of 234 mg/dL, and postprandial blood sugar level of 575 mg/dL. Serum electrolytes and other biochemical parameters were within normal limits. The patient tested negative for anti-HIV-1/2 antibodies, anti-HCV antibodies, and for Hepatitis B surface antigen. An ultrasonogram of the abdomen revealed a large well-defined hypoechoic lesion of size 13.8 × 11.6 × 12 cm, with dense internal echoes and debris in the left lobe of the liver suggestive of a large left-sided liver abscess with impending rupture. Pigtail catheterization carried out under local anaesthesia drained approximately 1000 ml of brownish yellow liquid pus, which was sent for routine microbiological investigations including Ziehl–Neelsen stain, wet-mount, and culture for bacteria and fungi (Figure 1(a)). Thereafter, cavity was irrigated, wound drainage was performed, and parenteral antibiotics (ceftriaxone, levofloxacin, and metronidazole) were administered to the patient empirically pending culture results. Simultaneously, glycemic control was optimized with insulin therapy.

Gram smear of the abscess drainage showed polymorphs with lanceolate-shaped Gram-positive cocci, lying in pairs, short chains, and small clusters (Figure 1(b)). The culture yielded pure growth of smooth, dome-shaped, and glistening alpha-haemolytic colonies on blood agar plates after overnight incubation at 37°C in 5–10% carbon dioxide, which on further incubation became flat with raised edges and central umbonation with carrom coin appearance (Figure 1(c)). No growth was observed on the MacConkey agar plate. The organism was catalase-negative, did not hydrolyse bile-esculin, was optochin sensitive, demonstrated bile solubility with 10% sodium deoxycholate solution, and was identified as *Streptococcus pneumoniae* [12].

Antimicrobial susceptibility testing using both disk diffusion and Etest (HiMedia, Mumbai, India) to determine, respectively, the zone diameter and minimum inhibitory concentrations (MICs) of various antibiotics showed the isolate to be susceptible to penicillin, ceftriaxone, levofloxacin, ertapenem, meropenem, clindamycin, chloramphenicol, vancomycin, and linezolid and resistant to erythromycin, doxycycline, and trimethoprim/sulfamethoxazole (Figure 1(d)) [13]. The MICs (*µ*g/ml) of penicillin, ceftriaxone, ertapenem, meropenem, clindamycin, vancomycin, and linezolid were 0.023, 0.016, 0.012, 0.023, 0.023, 0.5, and 0.19, respectively. Other routine microbiological investigations including blood culture did not reveal any significant finding. Amoebic serology was negative. Ziehl–Neelsen stain did not reveal any acid-fast bacteria. Wet-mount examination showed no trophozoites of *Entamoeba histolytica* or any fungal element. Antibiotic therapy with parenteral ceftriaxone was continued and clinical and radiological improvement was noted after two weeks, with decrease of abdominal pain and significant resolution of abscess. Parenteral ceftriaxone was administered for a total duration of 4 weeks, after which the patient was discharged, with advice of a 2-week course of oral cefixime. Clinical examination at four months and one-year of follow-up revealed no recurrence of infection or any new serious bacterial infection.

## 3. Discussion

*S. pneumoniae* is a colonizer in the nasopharynx of the human respiratory tract with 40–95% of infants and 1–10% of adults being colonized at any time [7]. It is the leading cause of pneumonia in children and is responsible for 30% of pneumonia cases in adults [7, 14]. The mortality rate for pneumococcal pneumonia can reach 11–40% even in developed countries [14]. In addition, it is responsible for a high burden of invasive pneumococcal diseases (consisting majorly of meningitis, sepsis, and bacteremia) in the general population with an annual incidence ranging from 23/100,000 to 188/100,000 [15, 16]. On an average, there are 4,100 cases of meningitis, 12,000 of bacteremia, 500,000 of pneumonia, and 7 million cases of otitis media due to *S. pneumoniae* annually in the United States alone [7]. Very rarely, unusual clinical manifestations of invasive pneumococcal diseases have been reported in literature consisting of pancreatic and liver abscess, aortitis, endocarditis, endophthalmitis, inguinal adenitis, arthritis and other osteoarticular infections, testicular and tubo-ovarian abscess, necrotizing fasciitis, pleural effusion, pyopneumothorax, mediastinitis with chest wall abscess, and multiple brain abscess [9, 17–20].

A review of relevant literature related to *S. pneumoniae* liver abscess revealed a mention of such entity only on 4 previous occasions reported as an individual case report in a single instance [8] and occurrence of a single case as part of microbiological spectrum of liver abscess cases in 3 others [9–11]. Almost similar to the present case, Gilardi and Dellepiane have described a patient with underlying diabetes mellitus who presented with liver abscess as the first manifestation of pneumococcal invasive disease, without respiratory symptoms and had a successful outcome on treatment with percutaneous drainage and systemic antibiotics [8]. Intra-abdominal abscesses such as liver abscesses generally develop as a result of abdominal surgery, intra-abdominal pathologies, or penetrating abdominal trauma [21, 22]. Rarely, abscesses can result from a distant focus of infection reaching the abdomen through infectious bacteremia, usually in individuals with poor oral hygiene or as a result of medical manipulation such as dental extraction, acupuncture, hemorrhoidectomy, or colonoscopy [22]. In the current patient, a careful history and subsequent clinical examination had revealed no evidence of any dental infection/caries, dental extraction or manipulation, no surgical history, and no history of bronchoscopy or any other respiratory tract manipulation in the recent past. The source of infection in the present case, thus, remains speculative similar to many other reports of PLAs where a definitive source of infection could not be determined. In a study, 80% of liver abscesses were cryptogenic in origin [5]. A notable feature of the present case was the absence of any significant systemic feature in the patient in the presence of a rapidly progressive and potentially fatal condition. The absence of systemic symptoms may perhaps be attributed to the immune dysfunction and impaired immune response, especially the reduction of cytokine responses such as interleukin-1 and interleukin-6, often seen in diabetic individuals [23].

As regards the antimicrobial susceptibility status, the Indian *S. pneumoniae* nonmeningeal isolates have largely retained their susceptibility to penicillin and third-generation cephalosporins with <1% of nonmeningeal pneumococcal isolates demonstrating penicillin and cefotaxime nonsusceptibility in a prospective laboratory-based surveillance conducted between January 2007 and July 2017 [24]. In another study on children younger than 5 years of age, pneumococcal resistance to trimethoprim/sulfamethoxazole, erythromycin, chloramphenicol, and penicillin was seen in 66%, 37%, 9%, and 8% of all invasive pneumococcal isolates, respectively [25].

## 4. Conclusion

Our report throws light on the occurrence of a clinically rare manifestation of a relatively commonly isolated pathogen, *S. pneumoniae,* in clinical samples. It is extremely important to establish an early diagnosis of *S. pneumoniae* infection for improved patient survival and favourable outcome. The correct identification of the pathogen was helpful in the timely resolution of infection following appropriate guided antimicrobial therapy. Therefore, clinicians must be able to recognize and manage unusual pneumococcal infections.

## Data Availability

The data used to support the findings of this study are included within the article.
